# The Type III Secretion System Effector SeoC of Salmonella enterica subsp. salamae and S. enterica subsp. arizonae ADP-Ribosylates Src and Inhibits Opsonophagocytosis

**DOI:** 10.1128/IAI.00704-16

**Published:** 2016-11-18

**Authors:** Dominic J. Pollard, Joanna C. Young, Valentina Covarelli, Silvia Herrera-León, Thomas R. Connor, Maria Fookes, Danielle Walker, Aurora Echeita, Nicholas R. Thomson, Cedric N. Berger, Gad Frankel

**Affiliations:** a MRC Centre for Molecular Bacteriology and Infection, Department of Life Sciences, Imperial College, London, United Kingdom; b Sección de Enterobacterias, Servicio de Bacteriología, Centro Nacional de Microbiología, ISCIII, Majadahonda, Spain; c Wellcome Trust Sanger Institute, Hinxton, Cambridge, United Kingdom; d The London School of Hygiene and Tropical Medicine, London, United Kingdom; University of Michigan

## Abstract

Salmonella species utilize type III secretion systems (T3SSs) to translocate effectors into the cytosol of mammalian host cells, subverting cell signaling and facilitating the onset of gastroenteritis. In this study, we compared a draft genome assembly of Salmonella enterica subsp. salamae strain 3588/07 against the genomes of S. enterica subsp. enterica serovar Typhimurium strain LT2 and Salmonella bongori strain 12419. S. enterica subsp. salamae encodes the Salmonella pathogenicity island 1 (SPI-1), SPI-2, and the locus of enterocyte effacement (LEE) T3SSs. Though several key *S*. Typhimurium effector genes are missing (e.g., *avrA*, *sopB*, and *sseL*), S. enterica subsp. salamae invades HeLa cells and contains homologues of S. bongori sboK and *sboC*, which we named *seoC*. SboC and SeoC are homologues of EspJ from enteropathogenic and enterohemorrhagic Escherichia coli (EPEC and EHEC, respectively), which inhibit Src kinase-dependent phagocytosis by ADP-ribosylation. By screening 73 clinical and environmental Salmonella isolates, we identified EspJ homologues in S. bongori, S. enterica subsp. salamae, and Salmonella enterica subsp. arizonae. The β-lactamase TEM-1 reporter system showed that SeoC is translocated by the SPI-1 T3SS. All the Salmonella SeoC/SboC homologues ADP-ribosylate Src E310 *in vitro*. Ectopic expression of SeoC/SboC inhibited phagocytosis of IgG-opsonized beads into Cos-7 cells stably expressing green fluorescent protein (GFP)-FcγRIIa. Concurrently, S. enterica subsp. salamae infection of J774.A1 macrophages inhibited phagocytosis of beads, in a *seoC*-dependent manner. These results show that S. bongori, S. enterica subsp. salamae, and S. enterica subsp. arizonae share features of the infection strategy of extracellular pathogens EPEC and EHEC and shed light on the complexities of the T3SS effector repertoires of Enterobacteriaceae.

## INTRODUCTION

Salmonella strains comprise a large group of foodborne bacterial pathogens of the gastrointestinal tract. Originally, the genus Salmonella was divided into seven subspecies (I, II, IIIa, IIIb, IV, V, and VI [1]) but is currently classified as two species, Salmonella bongori, previously subspecies V, and Salmonella enterica. The remaining subspecies have been renamed under the S. enterica species as enterica (I), salamae (II), arizonae (IIIa), diarizonae (IIIb), houtenae (IV), and indica (VI), which are further divided into over 2,600 serovars based on their O (oligosaccharide) and H (flagellar) antigens (2).

It is estimated that salmonellosis, the disease caused by consumption of contaminated food and water, is responsible for approximately 1.2 million cases and 450 fatalities per annum in the United States alone (3). Reservoirs of Salmonella spp. can be found in a range of domestic and wild animals, such as cattle, swine, poultry, and birds (4). In addition, exposure to exotic reptiles has been increasingly reported as a source of infection due to their growing popularity as pets (5). Salmonellae can cause a variety of conditions aside from the local diarrheal disease, including bacteremia, osteomyelitis, and enterocolitis (6).

The majority of salmonellosis cases observed in mammals and birds are a result of infections with S. enterica subsp. enterica. As a result, research into the remaining five S. enterica subspecies and S. bongori, often considered nonpathogenic commensals of cold-blooded vertebrates, is limited to date. Nevertheless, these other salmonellae do cause sporadic disease in mammals, with children and immunocompromised individuals most at risk (7–9). Several fatalities have been reported, and clinical evidence suggests that as a proportion of cases, those infections caused by non-enterica (I) subspecies are more likely to cause invasive extraintestinal disease (10).

Salmonellae encode two virulence-associated type III secretion systems (T3SSs) on Salmonella pathogenicity island 1 (SPI-1) and SPI-2, which are required for different stages of salmonellosis. T3SSs are macromolecular syringes, which translocate effectors into the membrane and cytosol of cells lining the gastrointestinal mucosae. The SPI-1 T3SS and its effectors are required for the initial infection process involving the invasion of nonphagocytic cell epithelium and M-cells and the stimulation of diarrhea (reviewed in reference 11). Once internalized, the bacterium resides within the specialized Salmonella-containing vacuole (SCV), where the SPI-2 is utilized for bacterial replication and systemic spreading of the infection. Recent reports have shown that Salmonella enterica subsp. salamae strains 1582 (serotype 58:d:z6), 1583 (serotype 47:b:1,5) (12), S1635, and S1296 (serovar Sofia, serotype 1,4,12,27:b:−) (13) carry genes similar to those carried by the locus of enterocyte effacement (LEE) pathogenicity island of the human pathogens enteropathogenic and enterohemorrhagic Escherichia coli (EPEC and EHEC, respectively) and the mouse pathogen Citrobacter rodentium, including the T3SS. Unlike members of S. enterica, S. bongori lacks SPI-2 and its SPI-1 locus appears to have acquired 11 genes not found in S. enterica subsp. enterica, 10 of which are homologues of T3SS effectors from EPEC and EHEC (14). For example, SboH from S. bongori shares sequential and functional homology to the antiapoptotic effector NleH1 (15). Furthermore, SboC shares 57% sequence identity to the EPEC antiphagocytic effector EspJ (14).

EPEC EspJ is able to ADP-ribosylate the kinase domain of Src, preventing the phosphorylation of the Fcγ-receptor-IIa (FcγRIIa) required for opsonophagocytosis (16, 17), and was the first example of a bacterial ADP-ribosyltransferase (ART) to target a mammalian tyrosine kinase. Mass spectrometry suggested a novel mechanism with coupled amidation and ADP-ribosylation of Src E310, a residue highly conserved throughout the kinase superfamily (18).

While producing a draft genome sequence for S. enterica subsp. salamae strain 3588/07, we found an *espJ/sboC* homologue, which we named SeoC, within a complex effector protein repertoire. The aim of this study was to determine the prevalence of *espJ*/*sboC/seoC* in representative clinical and environmental isolates from each of the S. enterica subspecies and to characterize their activity in relation to EspJ from EPEC, EHEC, and C. rodentium.

## MATERIALS AND METHODS

### Determination of the effector repertoire of S. enterica subsp. salamae strain 3588/07.

The whole genome of S. enterica subsp. salamae strain 3588/07 was sequenced using paired-end 454 FLX pyrosequencing using the Titanium chemistry from both 3-kb and 20-kb insert libraries. The read data were assembled using the 454/Roche Newbler assembly program into 138 contigs (*N*_50_ contig size, 173,757 bp; *N*_50_ scaffold size, 5,041,913 bp) representing 5,116,235 bp of sequence from 631,936 sequence reads representing 25× total coverage. We compared a draft assembly of S. enterica subsp. salamae (PATRIC accession no. GCA_000308035.1) against the genomes of S. enterica subsp. enterica serovar Typhimurium LT2 and S. bongori 12419. We performed all-against-all BLAST searches comparing each of these genomes against the S. enterica subsp. salamae assembly to identify regions with shared sequence similarity. We then visualized the BLAST searches using the Artemis Comparison Tool (19) and used known effectors in the Salmonella enterica serovar Typhimurium and S. bongori genomes to identify putative coding sequences for effectors in the S. enterica subsp. salamae assembly. We verified their presence based on a combination of direct examination of the nucleotide or amino acid sequence similarity and identification of their presence along with the same up- and/or downstream genes present in *S*. Typhimurium or S. bongori.

### Bacterial strains and growth conditions.

Bacterial strains used in this study can be found in Tables S1 and S2 in the supplemental material. Bacteria were routinely cultured in Luria-Bertani (LB) broth at 37°C, with tetracycline (6 μg/ml), ampicillin (100 μg/ml), or kanamycin (50 μg/ml) as appropriate.

Seventy-three strains were selected from the Collection of the Spanish National Laboratory for Salmonella. All the S. enterica strains were isolated in Spain, while S. bongori strains were from the National Salmonella Reference Laboratory at the Centers for Disease Control and Prevention, Atlanta, GA (see Table S2 in the supplemental material).

### Construction of S. enterica subsp. salamae mutants.

S. enterica subsp. salamae mutants (see Table S1 in the supplemental material) were created using the lambda red recombinase method (20). For S. enterica subsp. *salamae ΔseoC*, a PCR product was generated from S. enterica subsp. salamae including *seoC* and 500-bp flanking regions using primer pair 15 (see Table S3) and inserted into pGEMT vector by blunt-ended ligation. Inverse PCR with primer pair 16 was used to remove *seoC* coding sequence from this plasmid, and the kanamycin resistance cassette amplified from pKD4 using primer pair 18 was blunt-end ligated into the pGEMT backbone containing the *seoC* flanking regions. Primer pair 15 was used to amplify the kanamycin resistance cassette flanked by 500-bp flanking regions of *seoC*. For S. enterica subsp. *salamae ΔescN/ΔinvA/Δssav* mutants, the kanamycin resistance cassette from pKD4 was amplified with 50-bp flanking regions of *escN*, *invA*, or *ssaV* on either side, using primer pairs 20/21/22. PCR products from the two above methods were transformed by electroporation into electrocompetent S. enterica subsp. salamae 3558/07 expressing the lambda red genes from an arabinose-inducible promoter within the pKD46 plasmid (20). Clones were selected on 50-μg/ml kanamycin LB agar, cured by growth at 42°C, and verified by PCR and DNA sequencing using primer pairs 17/23/24/25 (for Δ*seoC/ΔescN/ΔinvA/Δssav*, respectively), 560 bp flanking the *seoC* gene, and primers for the kanamycin cassette.

### Plasmid construction.

Oligonucleotides for gene amplification and site-directed mutagenesis are shown in Table S3 in the supplemental material, plasmids are shown in Table S4, and the strains from which genes were amplified are shown in Table S1. Briefly, oligonucleotides were used to amplify the gene insert from genomic DNA, and products were purified using the Qiagen PCR purification kit according to the manufacturer's instructions. Target vectors and PCR products were digested for 1 h at 37°C with restriction enzymes before dephosphorylating the cut vector with calf intestinal phosphatase (New England BioLabs [NEB]) for 30 min at 37°C. Inserts and vectors were incubated together at a 3:1 molar ratio for 20 min at room temperature (RT) with T4 DNA ligase (NEB) before transformation into Top10 competent cells. Inserts were confirmed by colony PCR followed by DNA sequencing. Subcloning with EcoRI and HindIII restriction enzymes was used to “cut and paste” EPEC EspJ from pRK5-myc-EspJ_EPEC_ to pMALXE-EspJ_EPEC_. Site-directed mutagenesis was performed either by nonoverlapping inverse PCR followed by blunt-ended ligation or by overlapping inverse PCR and transformation using the QuikChange II site-directed mutagenesis kit (Agilent) according to the manufacturer's instructions.

### Screening and sequencing *seoC/sboC/espJ* orthologues among Salmonella isolates.

PCR was performed using *espJ* primers (primer pair 1 [see Table S3 in the supplemental material]) on bacterial boilates using a PuReTaq-Ready-To-Go PCR bead system (GE Healthcare). The amplification products of *espJ* were purified and sequenced using the same primers. Sequencing was performed on an ABI Prism 3730XL DNA analyzer (Applied Biosystems, Applera Hispania, S.A., Spain) using the *Taq* Dye Deoxy Terminator cycle sequencing kit (Applied Biosystems/Perkin-Elmer). Sequences analysis was performed with Lasergene v.5.0 software (DNAStar, Madison, WI, USA).

### Eukaryotic cell maintenance.

Cos-7, J774.A1, and HEK293 cells were maintained in Dulbecco's modified Eagle's medium (DMEM; Sigma-Aldrich) containing 4,500 mg/liter glucose supplemented with 10% (vol/vol) heat-inactivated fetal calf serum and 1% (vol/vol) GlutaMAX (Life Technologies) at 37°C and 5% CO_2_. HeLa cells were similarly maintained, only with 1,000 mg/liter glucose. Green fluorescent protein (GFP)-FcγIIa-stably transduced Cos-7 cells had the addition of 0.1 μg/ml of puromycin.

### Translocation assay.

The β-lactamase (TEM-1) translocation assay was performed as previously described (14). Briefly, 8 × 10^3^ HeLa/4 × 10^3^ J774.A1 cells were seeded per well of a 96-well plate (BD Falcon; catalogue no. 353948) 48 h prior to infection. Overnight cultures of S. enterica subsp. salamae strains were diluted 1:33 and grown for 2 h at 37°C with shaking before inducing TEM fusion expression from pCX340 (21) with 0.05 mM isopropyl-β-d-1-thiogalactopyranoside (IPTG). Cultures were then incubated to an optical density at 600 nm (OD_600_) of 1.8 (typically a further 30 min) and used to infect monolayers with a multiplicity of infection (MOI) of 100 for 1 h at 37°C, 5% CO_2_. The cell monolayers were washed with 100 μl Hanks' buffered salt solution (Gibco), supplemented with 20 mM HEPES and 3 mM probenecid (Sigma), pH 7.4, and developed as described previously (14). Fluorescence emission at 450 nm and 520 nm was measured using a FLUOstar Optima plate reader (excitation wavelength, 410 nm; 10-nm band-pass). The translocation rate was calculated as recommended in the Live Blazer FRET-B/G loading kit manual. Expression of the TEM-1 fusion proteins was analyzed by Western blotting using a mouse anti-β-lactamase antibody (QED Bioscience Inc.; data not shown).

### Protein overexpression.

EspJ homologues were cloned into pMALX(E) (22) backbone and Src 250–533_K295M/Y416F_ (Src_250–533KY_) and Src 250–533_K295M/Y416F/E310A_ (Src_250–533KYE_) were cloned into pGEX-KG to produce an N-terminally maltose binding protein (MBP)-tagged fusion and N-terminally glutathione *S*-transferase (GST)-tagged fusions, respectively. These vectors were transformed into BL21-STAR competent cells (Novagen), and overnight cultures were used to inoculate 1:100 of fresh LB broth. After growth to an OD_600_ of 0.6 at 37°C, protein expression from the T7 promoter was induced with 1 mM IPTG at 18°C overnight. Cells harvested at 4,500 relative centrifugal force (RCF) were resuspended in MBP lysis buffer (500 mM NaCl, 50 mM *N*-cyclohexyl-3-aminopropanesulfonic acid [CAPS], 5 mM dithiothreitol [DTT], 10% glycerol, pH 11) or GST lysis buffer (300 mM NaCl, 50 mM Na_2_HPO_4_, 5 mM DTT, 5% glycerol, pH 7.4) before cell disruption by sonication and clarification at 40,000 RCF.

### GST-Src 250–533 purifications.

Lysis supernatant was passed over a GSTrap 4B Sepharose column (GE Healthcare) to bind GST fusion proteins, and nonspecific interaction mixtures were washed with a 20× column volume of GST lysis buffer before being eluted with 20 mM reduced glutathione-containing elution buffer. Next, gel filtration in lysis buffer using a Superdex 200 10/300 gel filtration column (Invitrogen) was utilized to remove protein aggregates.

### *In vitro* ADP-ribosylation assay.

Five hundred microliters of lysis supernatant was incubated with 50 μl of amylose resin (Novagen) for 1 h at 4°C with rotation. After washing 3 times with MBP lysis buffer, 4 μg of GST-tagged Src_250–533KY_ or Src_250–533KYE_ and 10 μM 6-biotin-17-NAD^+^ (AMSBio) in phosphate-buffered saline (PBS), pH 7.4, were added. After 1 h, room-temperature (RT) Laemmli buffer was added and samples were boiled to stop the reaction.

### Western immunoblotting.

After separation by SDS-PAGE, the Bio-Rad Transblot semidry transfer cell was used to transfer samples to a polyvinylidene difluoride (PVDF) membrane for 1 h at 15 V. Membranes were blocked for 1 h at RT with 5% skim milk–PBS-Tween (PBST) (Sigma-Aldrich) before sequential incubation with primary and secondary antibodies in 1% skim milk-PBST for 1 h each with washing in between (see Table S5 in the supplemental material).

### Construction of a stably transduced Cos-7 GFP-FcγRIIa cell line.

GFP-FcγRIIa was cloned from pEGFP-FcγRIIa (23) into pMXs-IP (Invitrogen). HEK293 cells were seeded in a 6-well plate (Becton Dickinson) at 4.8 × 10^4^ cells per well 24 h before transfection. Cells were transfected with 500 ng pMX-IP–GFP-FcγRIIa, 400 ng of Moloney murine leukemia virus (MMLV) plasmid, and 100 ng of vesicular stomatitis virus G protein (VSV-G) plasmid using Lipofectamine (Invitrogen) according to the manufacturer's protocol. After 24 h, transfected cells were washed and fresh medium was added, allowing the cells to produce virions for a further 24 h when the GFP-FcγRIIa packaged virion-containing supernatant was collected. HEPES buffer was added to a final concentration of 20 mM, and the supernatant was filtered through a non-PVDF membrane before being added to a 70 to 90% confluent T25 flask of Cos-7 cells. After 24 h, the transduction of GFP-FcγRIIa was confirmed by fluorescence microscopy. Transduced cells were selected with 0.3 μg/ml puromycin for 1 week before fluorescence-activated cell sorting (FACS) using a BD FACS Aria III cell sorter and assessed using a BD FACS Fortessa III cell analyzer.

### Transfection of Cos-7 GFP-FcγRIIa cells.

Glass coverslips in 24-well tissue culture plates (Becton Dickinson) were seeded with 5 × 10^4^ cells per well 24 h prior to transfection. Cells were transfected using Genejuice transfection reagent (Novagen) at a 3:1 Genejuice/DNA ratio according to the manufacturer's instructions. DNA at 0.5 μg/well was transfected for 14 to 16 h before subjection to the opsonophagocytosis protocol.

### S. enterica subsp. salamae infection of J774.A1/HeLa cells.

J774.A1/HeLa cells were seeded on glass coverslips in a 24-well plate at 1.5 × 10^5^/7.5 × 10^4^ cells per well 24 h before infection, respectively. Overnight S. enterica subsp. salamae cultures were diluted 1:33 in Lennox-LB broth and incubated for 2.5 h at 37°C with shaking for 2.5 h to an OD_600_ of 0.8 before infection of macrophages with an MOI of 100. For complementation, genes were expressed from the pWSK29 backbone (24) using 0.05 mM IPTG 30 min prior to and during the infection. After 30 min of infection, bacterium-containing medium was removed and infected cells were challenged with opsonized beads as described below. For assessment of the bacterial invasion/internalization, S. enterica subsp. salamae expressing GFP from the pFPV25.1 plasmid (25) was used for infection. External bacteria were stained prepermeabilization using the BacTrace goat anti-CSA-1 antibody (Kirkegaard & Perry Laboratories [KPL]).

### Gentamicin protection invasion assay.

HeLa cells were infected as described above, washed with PBS after 60 min, and incubated with gentamicin-containing medium (200 μg/ml) for a further 60 min. Cells were then washed 5 times with PBS and lysed with 0.1% Triton X-100 before being plated in serial dilutions on LB agar to assess the number of CFU. Internalization is expressed as a percentage of the calculated inoculum.

### LDH release cytotoxicity assay.

J774.A1 cells were infected as described above, taking supernatant samples at 30 and 60 min. After centrifugation at 4,000 RCF followed by 20,000 RCF to remove mammalian and bacterial cells, supernatants were assessed for lactate dehydrogenase (LDH) release using the CytoTox 96 nonradioactive cytotoxicity assay (Promega) according to the manufacturer's protocol. Absorbance at 490 nm was measured using a medium-only control to calculate the net absorbance, and readings were normalized to uninfected controls.

### Bead opsonization and opsonophagocytosis assay.

Transfected cells were incubated for 2 h with serum-free DMEM (SF-DMEM). Meanwhile, 3.4-μm Sphero bovine serum albumin (BSA)-coated polystyrene beads (Spherotech) were opsonized for phagocytosis. Per coverslip, 10 μl (Cos-7 GFP-FcγRIIa cells) or 2.5 μl (J774.A1 cells) of bead slurry was washed in 1 ml of 20 mM 2-(*N*-morpholino)ethanesulfonic acid (MES)-8 mM HEPES before incubation with rotation with mouse anti-BSA primary antibody at room temperature (RT). After 1 h, opsonized beads were harvested and resuspended in 1 ml SF-DMEM (Cos-7 GFP-FcγRIIa cells) or serum-containing DMEM (J774.A1 cells) per coverslip before addition to transfected/infected cells. Beads were centrifuged onto the cells at 500 RCF for 5 min. Transfected cells were incubated at 4°C for 15 min before bead-containing medium was replaced with serum-containing DMEM, and phagocytosis was allowed to occur for 90 min. Infected macrophages were incubated with beads for 30 min at 37°C with 5% CO_2_. Infected/transfected cells were then washed once with cold tissue-grade PBS on ice before a 7-min staining of extracellular beads using donkey anti-mouse Alexa 488/RRX antibody on 0.2% BSA-PBS before washing twice with PBS and fixation with 3.7% paraformaldehyde (PFA)-PBS for 25 min.

### Immunofluorescence staining.

Fixed cells were neutralized with 50 mM NH_4_Cl-PBS for 15 min and permeabilized with 0.2% Triton X-100–PBS for 2 min. Nonspecific binding was blocked using 0.2% bovine serum albumin (BSA)-PBS for 15 min prior to incubation with primary antibodies (see Table S5 in the supplemental material). After 45 min, cells were washed 3 times with PBS before being blocked again and incubated with secondary antibodies.

### Quantification of bead internalization.

The internalization of beads associated with either transfected GFP-FcγRIIa Cos-7 cells or S. enterica subsp. salamae-infected J774.A1 cells was counted manually by immunofluorescence microscopy. Transfected cells were detected using chicken anti-myc antibody (Bethyl Laboratories), and bacteria were visualized with BacTrace goat anti-CSA-1 antibody (KPL).

### Accession number(s).

The S. enterica subsp. salamae 3588/07 genome sequencing reads from the 454 platform have been deposited in the Short Read Archive with accession no. ERR043066.

## RESULTS

### Effector repertoire of S. enterica subsp. salamae 3588/07.

The majority of sequenced S. enterica strains are from the enterica subspecies, but recently, a few genomes have been annotated from the non-enterica subspecies (12, 13, 26). We sequenced the genome of S. enterica subsp. salamae 3588/07 in order to compare its T3SS effector repertoire to other Salmonella strains. Of the seven S. enterica subsp. salamae isolates, four were from a water bath area which strain 3588/07 was selected to represent. The draft genome sequence is represented by 140 contigs and is 5,116,236 bp in size. In agreement with previously sequenced S. enterica subsp. salamae strains (12, 13), S. enterica subsp. salamae 3588/07 carries the SPI-1, SPI-2, and LEE T3SSs, including the signature LEE region proteins encoded by *escN*, the ATPase required for EPEC/EHEC T3SS effector translocation (27), and the translocon proteins EspA/B/D. The EPEC/EHEC outer membrane adhesin intimin was present along with six translocated effectors, including the intimin receptor Tir (28). However, the LEE-encoded effectors Map, EspG, and EspH were missing (29). The entire effector repertoire of S. enterica subsp. salamae 3588/07 (Table 1) reveals that several key effectors found in *S*. Typhimurium are not present, including the anti-inflammatory effectors AvrA (29) and GogB (30) and the deubiquitinase SseL (31) (Table 1). In particular, only a short, 189-bp fragment of the *sopB* gene, which is considered a core effector with regard to Salmonella virulence, is found in the S. enterica subsp. salamae 3588/07 genome, compared to the 1,686 bp in *S*. Typhimurium. S. enterica subsp. salamae 3588/07 also encodes the effectors SteA, SteB, SteC, SopE, SspH1, and SseK2, which have not been previously found in S. enterica subsp. salamae strains (12). In addition, the S. bongori effectors SboK and SboC are also present in S. enterica subsp. salamae 3588/07. SboC is homologous to the EPEC, EHEC, and C. rodentium antiphagocytic effector EspJ; we named the S. enterica subsp. salamae homologue SeoC (Salmonella enterica outer protein C).

**TABLE 1:** Comparison of Salmonella T3SS effectors^*a*^

Secretion system or gene	Genomic location	Secreting system(s)	Presence or absence of T3SS effector system or protein in Salmonella strain^*b*^:					
			S. enterica subsp. salamae 3588/07	S. enterica subsp. salamae 1582	S. enterica subsp. salamae 1583	S. enterica subsp. arizonae CDC346-86	S. bongori 12419	*S*. Typhimurium LT2
Secretion systems								
SPI-1 T3SS			+	+	+	+	+	+
SPI-2 T3SS			+	+	+	+	−	+
Gene name								
*avrA*	SPI-1	SPI-1	−	−	−	−	+	+
*sboC*/*seoC*	ROD	SPI-1	+	+	+	+	+	−
*sipA* (*sspA*)	SPI-1	SPI-1	+	+	+	+	+	+
*sipB* (*sspB*)	SPI-1	SPI-1	+	+	+	+	+	+
*sipC* (*sspC*)	SPI-1	SPI-1	+	+	+	+	+	+
*sopA*	BB	SPI-1	+	4	5	−	−	+
*sopD*	BB	SPI-1	+	+	+	+	+	+
*sopE*	fSopE, fSE12	SPI-1	+	?	?	−	−	−
*sopE2*	BB	SPI-1	+	+	+	+	+	+
*sptP*	SPI-1	SPI-1	+	+	+	+Ψ	+Ψ	+
*sopB* (*sigD*)	SPI-5	SPI-1	F	?	?	+	+	+
*slrP*	BB	SPI-1 and -2	−	−	−	+	+	+
*sspHI*	ROD	SPI-1 and -2	+	?	?	−	−	+@
*gogB*	fGifsy-1	SPI-2	−	−	−	−	−	+
*pipB*	SPI-5	SPI-2	−	−	−	−	−	+
*spvB*	pSLT plasmid	SPI-2	−	−	−	+	−	+
*sseI* (*srfH*)	fGifsy-2	SPI-2	−	−	−	+	−	+
*sseK1*	ROD	SPI-2	−	?	?	+	−	+
*sseL*	BB	SPI-2	−	?	?	+	−	+
*sspH2*	SPI-12	SPI-2	−	−	−	+	−	+
*pipB2*	ROD	SPI-2	+	+	+	−	−	+
*sifA*	BB	SPI-2	+	+	+	+	−	+
*sifB*	BB	SPI-2	+	+	+	+	−	+
*sopD2*	BB	SPI-2	+	+	?	+	−	+
*spiC* (*ssaB*)	SPI-2	SPI-2	+	+	+	+	−	+
*sseF*	SPI-2	SPI-2	+	+	+	+	−	+
*sseG*	SPI-2	SPI-2	+	+	+	+	−	+
*sseJ*	BB	SPI-2	+	+	+	+	−	+
*sseK2*	ROD	SPI-2	+	?	?	−	−	+
*steA*	BB	SPI-2	+	?	?	−	−	+
*steB*	BB	SPI-2	+	?	?	−	−	+
*steC*	BB	SPI-2	+	?	?	−	−	+
*sseK3*	fSE20	−	−	?	?	−	−	−&
*sboA*	fSB100	S. bongori T3SS	−	?	?	−	+	−
*sboB*	ROD	S. bongori T3SS	−	?	?	−	+	−
*sboD*	ROD	S. bongori T3SS	−	?	?	−	+	−
*sboE*	Degenerate f	S. bongori T3SS	−	?	?	−	+Ψ	−
*sboF*	fSB100	S. bongori T3SS	−	?	?	−	+	−
*sboG*	fSB101	S. bongori T3SS	−	?	?	−	+	−
*sboH*	BB	S. bongori T3SS	−	2	−	−	+	−
*sboI*	ROD	S. bongori T3SS	−	−	−	−	+	−
*sboJ*	ROD	S. bongori T3SS	−	−	−	−	+	−
*sboK*	ROD	S. bongori T3SS	+	−	+	−	+	−
*sboL*	ROD	S. bongori T3SS	−	−	2	−	+	−

a Symbols and abbreviations: ?, presence not confirmed in the work of Desai et al. (12); +, present; −, absent; Ψ, pseudogene; BB, chromosomal backbone; ROD, region of difference/genomic island; ^@^, of limited distribution in *S*. Typhimurium (30); &, carried on phage ST64B of other *S*. Typhimurium strains (23); F, *sopB* was missing in S. enterica subsp. salamae, but there is a fragment of approximately 180 bp that has 58 to 74% sequence similarity to *sopB* in the same genomic position found in S. bongori.

b Numbers 2, 4, and 5 indicate the number of loci at which the gene is predicted in the genome.

### Prevalence of *sboC/seoC* in S. bongori and S. enterica spp.

We investigated the prevalence of *seoC* across the Salmonella genus by screening 73 clinical and environmental Salmonella isolates by PCR. Strain selection was based upon belonging to different species/subspecies of Salmonella and to the most frequent serotypes within each species/subspecies and, if possible, being isolated from human/nonhuman sources (see Table S2 in the supplemental material). All tested S. bongori isolates were *sboC* positive. Of the S. enterica isolates, *seoC* was found in 4 out of 7 isolates belonging to subspecies salamae and in 8 out of 9 isolates belonging to subspecies arizonae but not in any isolates of subspecies enterica, diarizonae, or houtenae (see Table S2).

**FIG 1:**
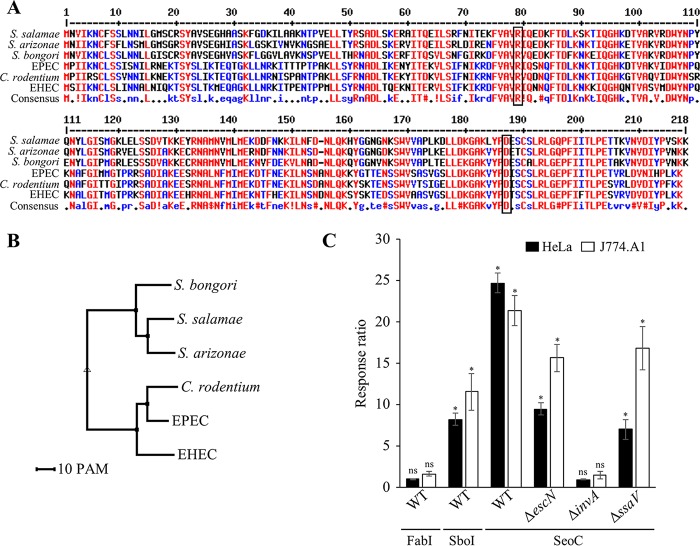
Sequence alignment of EspJ homologues and protein translocation. (A) Sequence alignment of the EspJ homologues with consensus sequence. Residues are colored red for high consensus, blue for low consensus, and black for neutral consensus. The conserved residues R79 and D187 crucial for EPEC EspJ activity are highlighted with a black box. (B) Phylogenetic analysis of the EspJ homologues showing separation of EPEC, EHEC, and C. rodentium homologues from the Salmonella homologues. A scale for point accepted mutations (PAM) is displayed. (C) S. enterica subsp. salamae SeoC is translocated via the SPI-1 (Δ*invA*), but not via the SPI-2 (Δ*ssaV*) or LEE (Δ*escN*), T3SS during S. enterica subsp. salamae infection of HeLa and J774.A1 cells. The T3SS effector SboI and FabI were used as positive and negative controls, respectively. Results are averages from three experiments, each in quadruplicate wells. The error bars represent standard errors of the means. Statistical analyses were performed with GraphPad Prism software using a one-way analysis of variance followed by Bonferroni posttest (*, *P* < 0.01). Asterisks represent significance compared to uninfected cells; ns, not significant.

Amino acid sequence alignment (Fig. 1A) revealed that SeoC from S. enterica subsp. arizonae and that from S. enterica subsp. salamae share 83% sequence identity and 77%/78% identity, respectively, to SboC from S. bongori. Between 56% and 58% sequence identity is shared between the Salmonella SeoC/SboC and EspJ from EPEC, EPEC, and C. rodentium (see Table S6 in the supplemental material). Sequence variation was concentrated at the N-terminal 50 amino acids with only around 35% sequence identity between the *E. coli/C. rodentium* and Salmonella homologues (see Table S6). Typically, this region harbors a 20- to 30-residue secretion signal, required for specific targeting to the correct secretion system. Phylogenetic analysis revealed that the Salmonella and the *E. coli/C. rodentium* homologues fall on separate branches in the tree (Fig. 1B). However, residues R79 and D187, required for EPEC EspJ ART activity, are preserved in all EspJ/SeoC homologues, suggesting a conserved catalytic function (Fig. 1A, black box).

### S. enterica subsp. salamae SeoC is translocated by the SPI-1 T3SS.

In order to determine through which T3SS the S. enterica subsp. salamae SeoC is translocated, translocation assays were performed using wild-type (WT) S. enterica subsp. salamae and LEE (Δ*escN*), SPI-1 (Δ*invA*), and SPI-2 (Δ*ssaV*) T3SS nonfunctional mutants expressing SeoC fused to the TEM-1 reporter. The housekeeping/cytosolic protein FabI fused to TEM-1, used as a negative control, was not translocated when expressed in WT S. enterica subsp. salamae, while the S. bongori T3SS effector SboI, which was used as a positive control, was translocated into both HeLa cells and J774.A1 macrophages. SeoC-TEM was translocated into both HeLa cells and J774.A1 macrophages following infection with WT S. enterica subsp. salamae, and to a lesser extent from the Δ*escN* and Δ*ssaV* mutants. In contrast, no translocation was seen from the Δ*invA* strain (Fig. 1C), suggesting that SeoC is an SPI-1 effector.

### S. enterica subsp. salamae invades phagocytic and epithelial cells independently of SeoC.

**FIG 2:**
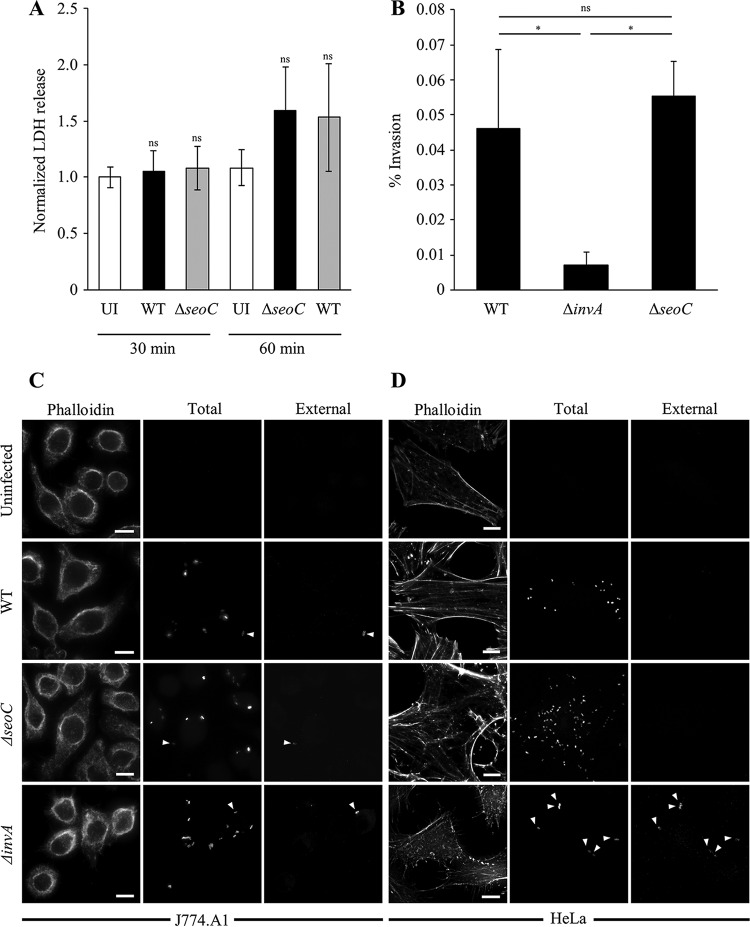
Internalization of S. enterica subsp. salamae 3588/07 during infection of HeLa and J774.A1 cells. (A) WT and Δ*seoC S. enterica* subsp. salamae strains do not cause cell lysis compared to uninfected J774.A1 cells 30 or 60 min postinfection. Data represent the averages from three experiments, each in triplicate. UI, uninfected; ns, not significant. (B) Gentamicin protection assay of HeLa cells infected with WT S. enterica subsp. salamae or Δ*seoC* or Δ*invA* mutant. There was no significant difference between S. enterica subsp. salamae WT and Δ*seoC* strain invasiveness, while the Δ*invA* strain was significantly less invasive than both WT and Δ*seoC* strains. Results are the mean ± standard error of the mean from three or more independent experiments. Statistical analyses were performed with GraphPad Prism software using a one-way analysis of variance followed by Bonferroni posttest (*, *P* < 0.01; ns, not significant). (C and D) J774.A1 (C) and HeLa (D) cells were infected with WT S. enterica subsp. salamae or the Δ*seoC* or Δ*invA* mutant expressing GFP. External bacteria were stained with anti-CSA-1 antibody before cell permeabilization. All strains were internalized by J774.A1 macrophages (C). WT and Δ*seoC* strains invaded HeLa cells, while the Δ*invA* strain was not invasive (D). Arrowheads indicate external bacteria. Bars, 10 μm.

S. enterica subsp. salamae is mainly isolated from extraintestinal infections (10). We therefore aimed to investigate the invasion potential of S. enterica subsp. salamae and any involvement of SeoC in this process, considering that EspJ has an antiphagocytic activity, albeit of opsonized particles (16). Salmonella expressing GFP (25) was used to infect HeLa cells and J774.A1 macrophages. Lactate dehydrogenase (LDH) release, indicative of cell lysis, measured 30 and 60 min after infection of J774.A1 with WT and Δ*seoC S. enterica* subsp. salamae, revealed no significant increase over uninfected cells (Fig. 2A), suggesting that these strains are not cytotoxic. Gentamicin protection invasion assays showed that while S. enterica subsp. salamae Δ*invA* was significantly less invasive into HeLa cells, no significant difference was seen between the WT and S. enterica subsp. salamae Δ*seoC* strains (Fig. 2B). Extracellular bacterial staining, using the anti-CSA-1 antibody prepermeabilization, confirmed that S. enterica subsp. salamae and S. enterica subsp. salamae Δ*seoC* invaded both phagocytic and epithelial cells (Fig. 2C and D). The SPI-1 T3SS-deficient Δ*invA* strain was still internalized by J774.A1 macrophages but unable to invade HeLa cells. Together, these data show that, similarly to EspJ, SeoC does not play a role in inhibition of *cis*-phagocytosis (uptake of nonopsonized bacteria).

### SeoC ADP-ribosylates Src E310.

**FIG 3:**
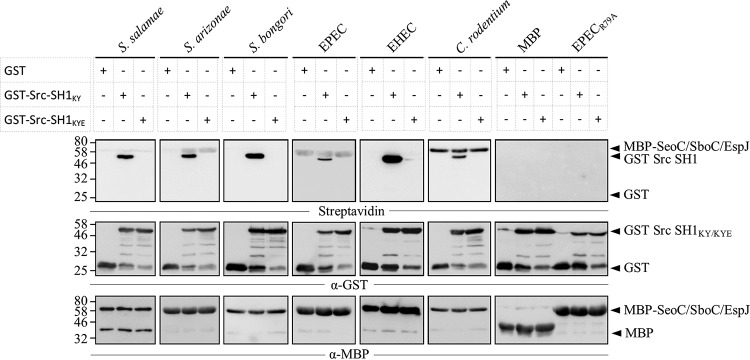
Salmonella and E. coli EspJ homologues ADP-ribosylate Src E310 *in vitro*. MBP-tagged EspJ homologues bound to amylose resin were incubated with Src-SH1_KYE_ and 6-biotin-17-NAD^+^ (NAD-biotin) for 1 h and analyzed by Western blotting. ADP-ribosylated proteins were detected using a streptavidin-horseradish peroxidase conjugate (top row), while the anti-GST antibody revealed GST-tagged Src-SH1_KY/KYE_ (middle row) and the anti-MBP antibody revealed MBP-tagged SeoC/SboC/EspJ (bottom row). The proteins detected by Western blotting are indicated with arrowheads on the right. All EspJ homologues were able to ADP-ribosylate GST-Src-SH1_KY_ but not GST-Src-SH1_KYE_. Numbers at left are molecular masses in kilodaltons.

We have previously reported that EPEC EspJ inhibits Src by ADP-ribosylation of E310 within its protein kinase domain (SH1) (17). In order to determine if SeoC from S. enterica subsp. salamae and S. enterica subsp. arizonae, SboC from S. bongori, and EspJ from EHEC and C. rodentium share this activity, we performed an *in vitro* ADP-ribosylation assay. Purified MBP-tagged effectors and GST-tagged Src-SH1_K295M/Y416F_ (Src-SH1_KY_; K295M, kinase dead; Y416F, autophosphorylation mutant) were incubated with NAD-biotin and then analyzed by Western blotting. This revealed that SeoC, SboC, and EspJ ADP-ribosylated GST-Src-SH1_KY_ (Fig. 3, lower band, streptavidin panel) and also had various levels of autoactivity (Fig. 3, upper band, streptavidin panel). No ADP-ribosylation was detected using the negative control GST or when GST-Src was incubated with MBP-EspJ-EPEC_R79A_ (ART catalytic mutant) or MBP. Importantly, we detected no ADP-ribosylation of Src mutated at residue E310 (Src-SH1_KYE_), suggesting that the target residue is shared between SeoC, SboC, and EspJ.

### Ectopic expression of SeoC inhibits FcγRIIa-mediated phagocytosis.

**FIG 4:**
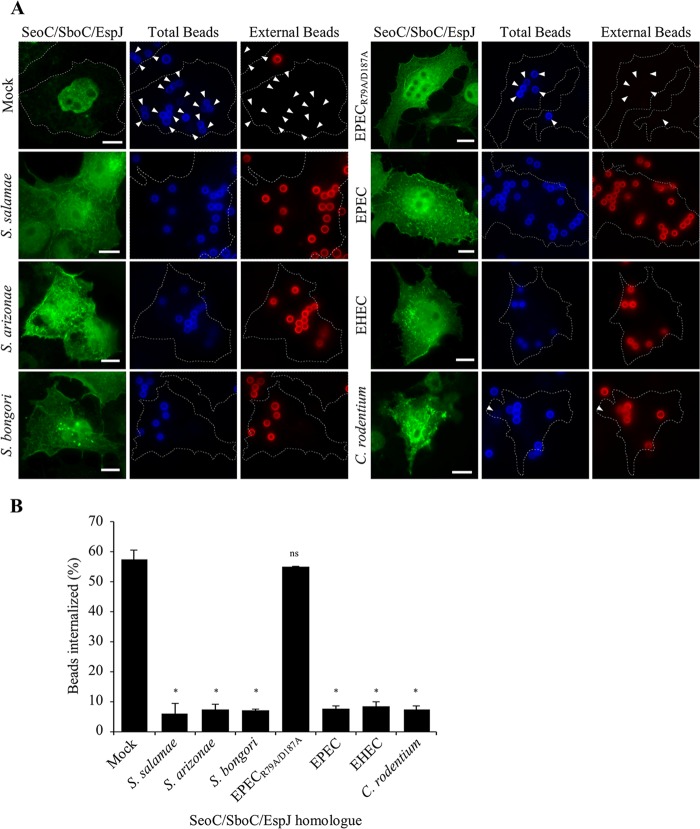
Ectopic expression of Salmonella EspJ homologues inhibits FcγRIIa-mediated phagocytosis. Cos-7 cells stably expressing GFP-FcγRIIa and transfected with plasmids encoding Myc-tagged SeoC/SboC/EspJ homologues were challenged with IgG-opsonized 3-μm beads. (A) Staining pre- and postpermeabilization revealed external beads (red), total beads (blue), and SeoC/SboC/EspJ-transfected cells (green). Representative immunofluorescence images of pRK5-SeoC/SboC/EspJ-transfected cells are shown. Arrowheads indicate the internalization of beads in SeoC/SboC/EspJ-transfected cells; dotted lines indicate the cell outlines. Bars, 10 μm. (B) Total/external cell-associated beads were counted for EspJ-transfected cells, revealing inhibition of bead phagocytosis by all SeoC/SboC/EspJ homologues compared to mock-transfected (Mock) or EPEC EspJ_R79A/D187A_ (ART mutant)-transfected cells. Results are the mean ± standard error of the mean from three independent experiments. Statistical analyses were performed with GraphPad Prism software using a one-way analysis of variance followed by Bonferroni posttest (*, *P* < 0.01; ns, not significant).

Src kinase is involved in the regulation of many cellular processes, including cell proliferation and differentiation, cell motility, and phagocytosis (reviewed in reference 32). Src phosphorylates the cytoplasmic immunotyrosine activation motifs (ITAMs) of FcγRIIa, allowing the recruitment of Syk kinase and initiating the signaling cascade for phagocytic actin remodeling. To assess the inhibition of phagocytosis by the different SeoC/SboC/EspJ homologues, we generated a Cos-7 cell line stably expressing FcγRIIa. The cells were then transfected with the different SeoC/SboC/EspJ homologues before being challenged with IgG-opsonized beads. The internal/external localization of cell-associated beads in transfected cells was observed by immunofluorescence (Fig. 4A). Mock-transfected cells or cells transfected with EPEC EspJ_R79A/D187A_ (ART mutant) were used as negative controls. Immunofluorescence analysis showed that all the SeoC/SboC/EspJ homologues reduced bead internalization from 60% in mock- and EspJ_R79A/D187A_-transfected cells to below 10% (Fig. 4B). Thus, the ADP-ribosylation of Src appears inhibitory, and each effector is capable of inhibiting opsonophagocytosis, independently of other effector proteins.

### SeoC inhibits FcγR-mediated phagocytosis during Salmonella infection.

**FIG 5:**
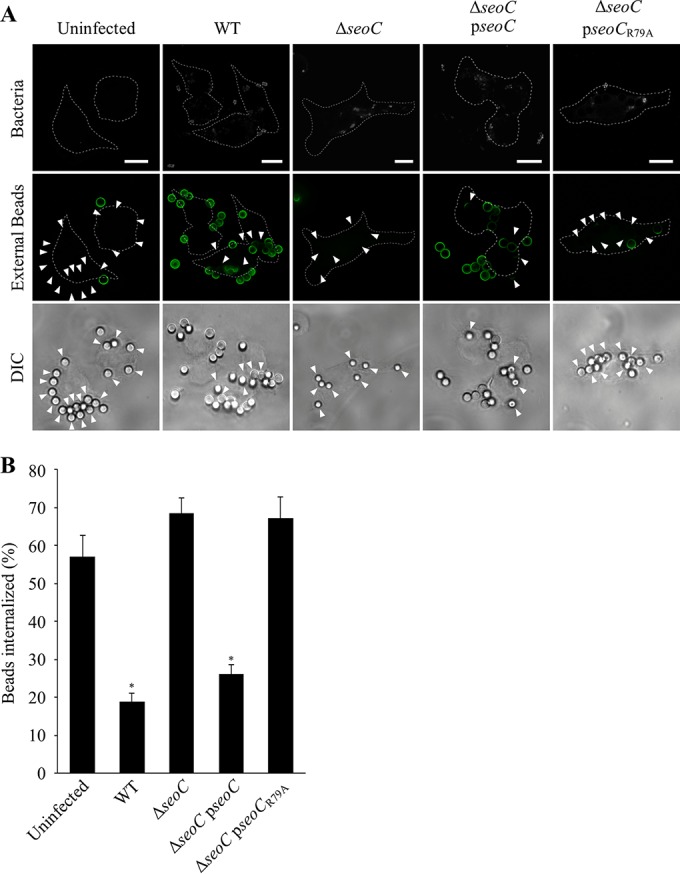
SeoC inhibits FcγRIIa-mediated phagocytosis during S. enterica subsp. salamae infection of J774.A1 macrophages. J774.A1 macrophages were infected with WT S. enterica subsp. salamae 3588/07 or Δ*seoC* mutant with or without complementation by pWSK29-SeoC/SeoC_R79A_ (p*seoC*/p*seoC*_R79A_) before challenge with IgG-opsonized beads. (A) Representative immunofluorescence images are shown with external beads stained prepermeabilization (green), total beads shown using differential interference contrast (DIC), and bacteria stained with anti-CSA-1 (white). Macrophage outlines are shown with dotted lines. Arrowheads indicate the internalization of cell-associated beads by infected macrophages. Bar, 10 μm. (B) External and total cell-associated beads were counted, revealing that infection with WT S. enterica subsp. salamae 3588/07 reduced internalization from 57% of beads for uninfected cells to 18.8%. Deletion of *seoC* removed the inhibition of phagocytosis, while complementation with WT *seoC* but not *seoC*_R79A_ (ART mutant) was able to restore the inhibition. Results are the mean ± standard error of the mean from three independent experiments. Statistical analyses were performed with GraphPad Prism software using a one-way analysis of variance followed by Bonferroni posttest (*, *P* < 0.01).

To confirm the inhibitory activity of SeoC during infection, J774.A1 macrophages were infected with WT S. enterica subsp. salamae, S. enterica subsp. salamae Δs*eoC*, and S. enterica subsp. salamae Δs*eoC* complemented with plasmids carrying *seoC* or *seoC*_R79A_ and then challenged with IgG-opsonized beads. Immunofluorescence revealed that macrophages infected with WT S. enterica subsp. salamae displayed reduced phagocytosis of cell-associated beads (<18%) compared to uninfected cells (57%) or cells infected with S. enterica subsp. salamae Δ*seoC* (68%) (Fig. 5A and B). S. enterica subsp. salamae Δ*seoC* complemented with *seoC* restored inhibition of phagocytosis (26%), whereas the *seoC*_R79A_ strain had a level of phagocytosis similar to that of the Δ*seoC* mutant, confirming that ADP-ribosylation activity of SeoC is required for inhibition of phagocytosis.

## DISCUSSION

In this study, genomic sequencing revealed the effector repertoire of the SPI-1, SPI-2, and LEE T3SSs of S. enterica subsp. salamae 3588/07, a crucial determinant for bacterial infection strategies and host specificities. For example, the absence of *sseJ* from Salmonella enterica serovar Typhi was linked to reduced cytotoxicity in *in vitro* infection models (33). Similarly, a Salmonella enterica serovar Typhimurium *slrP* mutant caused a colonization defect in mice but had no effect in calves (34). Despite its importance in *S*. Typhimurium, SlrP is not found in S. enterica subsp. salamae and is observed only as a pseudogene in many other Salmonella strains. We observed six effector genes not previously seen in S. enterica subsp. salamae. These were *steA*, *steB*, and *steC*, with unknown function; *sseK2*, which is similar in sequence to the EPEC/EHEC anti-inflammatory effector NleB (35); the NF-κB inhibitor with E3 ligase activity *sspH1* (36); and the guanine nucleotide exchange factor (GEF) gene *sopE*. In *S*. Typhimurium, a *sopE*, *sopE2*, and *sopB* triple mutant is noninvasive (37). Both *sopE* and *sopE2* are present in S. enterica subsp. salamae 3558/07, but *sopB*, which contributes to invasion, fluid secretion, SCV development, and intracellular survival (38, 39), and *avrA*, which also supports intracellular survival (29), were absent. Desai et al. identified paralogues of S. bongori effector SboH in S. enterica subsp. salamae 1582, SboK and SboL in S. enterica subsp. salamae 1583, and SboC in both strains (12). S. enterica subsp. salamae 3588/07 possesses SboK, which has no known function but is predicted to have a domain organization similar to SlrP, and SboC, the predicted homologue of EPEC/EHEC EspJ. Strain 3588/07 was selected to represent the four of seven S. enterica subsp. salamae isolates originating from water bath areas. Differences in its effector repertoire from those of other S. enterica subsp. salamae strains highlight that the remaining six isolates are likely to possess unique subsets of effectors, which could be revealed by further genome sequencing.

PCR screening showed that *sboC/seoC* was present in S. bongori (6 from 6), S. enterica subsp. salamae (4 from 7), and S. enterica subsp. arizonae (8 from 9) isolates, but none of the S. enterica subsp. diarizonae and S. enterica subsp. houtenae isolates tested. S. bongori SboC is known to be translocated by the T3SS (14), and this study shows that S. enterica subsp. salamae SeoC is mainly a substrate of the SPI-1 T3SS. SeoC did, however, inhibit phagocytosis during the infection of murine macrophages, as did the ectopic expression of all tested SeoC/SboC/EspJ homologues within a GFP-FcγRIIa-expressing Cos-7 cell line. It is possible for effector proteins to be substrates of more than one secretion system at different stages of infection, as is the case for SlrP (40). For SeoC, this could explain the reduction in translocation in LEE and SPI-2 T3SS mutant strains, which would be interesting to investigate using secretion assays specific to each T3SS.

Salmonella and E. coli are members of the same Enterobacteriaceae family with common ancestry over 100 million years ago (41). While pathogenic E. coli types such as EPEC and EHEC possess a LEE-encoded T3SS and have adapted an extracellular lifestyle, the majority of Salmonella strains have SPI-1 and SPI-2 T3SSs for invasion and intracellular replication, respectively. The SPI-1 T3SS was acquired prior to the divergence of S. bongori and S. enterica, after which the SPI-2 T3SS was introduced, before separation into further subspecies (42). Pathogenicity islands are generally acquired by horizontal gene transfer but can also be carried in integrative elements such as cryptic prophages (37). Horizontal gene transfer has allowed the diversification and specialization of Salmonella infection strategies, and the distribution of the SeoC/SboC/EspJ homologues in Salmonella and *E. coli/C. rodentium* is a good example of this.

The presence of both SPI-1 and SPI-2 T3SSs in S. enterica subsp. salamae and S. enterica subsp. arizonae suggests an intracellular lifestyle, while the identification of a LEE-encoded T3SS in S. enterica subsp. salamae could promote the opposite. It has been reported that S. enterica subsp. arizonae and S. enterica subsp. diarizonae are internalized poorly by J774.A1 macrophages (43). Additionally, from a variety of Salmonella serovars isolated from crocodiles, only subspecies enterica and not salamae or diarizonae displayed invasive phenotypes in a mouse model system (44). In fact, the presence of both SPI-1 and SPI-2 does not necessarily confer invasiveness *in vivo*, as although the transfer of SPI-2 to S. bongori increased intracellular persistence in cell culture, systemic pathogenicity in a murine model was not possible (45). We showed that S. enterica subsp. salamae can invade both J774.A1 and HeLa cells in a SeoC-independent manner and lacks the actin-rich pedestals characteristic of the LEE T3SS. This observation is interesting considering the lack of *sopB* and *avrA* in S. enterica subsp. salamae, which are key effectors for *S*. Typhimurium invasiveness and intracellular survival. For further characterization of the salamae subspecies, it will be useful to investigate invasiveness into other epithelial and macrophage cell lines. The SeoC-independent invasiveness of S. enterica subsp. salamae shows that SeoC, like EspJ, does not have a role in the inhibition of *cis*-phagocytosis.

Inhibition of phagocytosis by EPEC EspJ is mediated by amidation and ADP-ribosylation of the kinase domain of Src (17). Many bacterial toxins possess ART activity, and several T3SS effector proteins with ART activity have been identified, including Pseudomonas syringae HopF2, Pseudomonas aeruginosa ExoS and ExoT, and Salmonella SpvB. HopF2 shares 20 to 25% sequence identity with the SeoC/SboC/EspJ homologues and ADP-ribosylates multiple mitogen-activated protein kinases (MAPKs) and RIN4, inhibiting plant pathogen-associated molecular pattern (PAMP)-triggered defenses (46, 47). The catalytic activity of SpvB is essential for virulence of *S*. Typhimurium in mice (48), via the modification of actin, a target for many ADP-R toxins, including Clostridium botulinum C2, Clostridium perfringens iota toxin, and the Clostridium difficile toxin (CDT) (49). Of the non-enterica subspecies, *spvB* is present only in S. enterica subsp. arizonae, making SeoC and SboC the first T3SS translocated ARTs identified in S. enterica subsp. salamae and S. bongori. We showed that all the EspJ homologues ADP-ribosylate E310 within the kinase domain of Src, inhibiting Src-dependent phagocytosis signaling. ExoT also inhibits phagocytosis and has many observed targets, including Ras, ezrin/radixin/moesin (ERM) proteins, and Rab5 (50). As Src E310 is highly conserved throughout the kinase superfamily, the SeoC/SboC/EspJ homologues may, too, have additional targets, the discovery of which is vital for uncovering the ultimate role of these effectors. This could be pursued using proteomic mass spectrometry after *Salmonella/E. coli* infections to identify ADP-ribosylated proteins or changes to the phosphoproteome indicating the inactivation of target kinases.

The presence of SeoC/SboC/EspJ homologues in a subset of bacteria with stark differences in their infection strategies displays the importance of horizontal gene transfer for shaping the complex T3SS effector repertoire of Enterobacteriaceae. It would be interesting to study the consequences of expressing SeoC/SboC in *S*. Typhimurium, which lacks this translocated enzyme, using the mouse model of salmonellosis. In combination with powerful mass spectrometry analysis, this will help uncover the ultimate impact of these homologues on Salmonella infection strategies.

## Supplementary Material

Supplemental material
