# Supplementary material for: The Type III Secretion System Effector SeoC of Salmonella enterica subsp. salamae and S. enterica subsp. arizonae ADP-Ribosylates Src and Inhibits Opsonophagocytosis
Source: Infect Immun. 2016 Nov 18;84(12):3618–28. doi: 10.1128/IAI.00704-16 (PMC5116738; doi:10.1128/IAI.00704-16)
Supplement: Supplemental material [file supp_84_12_3618__index.html]

Supplemental material 

# The Type III Secretion System Effector SeoC of Salmonella enterica subsp. salamae and S. enterica subsp. arizonae ADP-Ribosylates Src and Inhibits Opsonophagocytosis

## Supplemental material

**Files in this Data Supplement:**

- Supplemental file 1 -

  Table S1. Bacterial strains. Table S2. List of *Salmonella* strains analyzed in the *espJ* homologue screen. Table S3. Primers. Table S4. Plasmids. Table S5. Primary and secondary antibodies and reagents. Table S6. Percent sequence identity between SeoC/SboC/EspJ.

  PDF, 948K
