# Supplementary material for: The Type III Secretion System Effector SeoC of Salmonella enterica subsp. salamae and S. enterica subsp. arizonae ADP-Ribosylates Src and Inhibits Opsonophagocytosis
Source: Infect Immun. 2016 Nov 18;84(12):3618–28. doi: 10.1128/IAI.00704-16 (PMC5116738; doi:10.1128/IAI.00704-16)
Supplement: Supplemental material [file IAI.00704-16_zii999091905so1.pdf]

Table S1: Bacterial strains

Genus	Species	Subspecies	Pathotype	Mutation	O antigen	H antigen(s)	Strain
Salmonella	*enterica*	*salamae*	-	-	13,22	z10:z6	3588/07
Salmonella	*enterica*	*salamae*	-	Δ*seoC*	13,22	z10:z6	3588/07
Salmonella	*enterica*	*salamae*	-	Δ*escN*	13,22	z10:z6	3588/07
Salmonella	*enterica*	*salamae*	-	Δ*invA*	13,22	z10:z6	3588/07
Salmonella	*enterica*	*salamae*	-	Δ*ssaV*	13,22	z10:z6	3588/07
Salmonella	*enterica*	*arizonae*	-	-	62	z36:-	SARC6
Salmonella	*bongori*	-	-	-	48	z36:−	CEIM46082
Escherichia	*coli*	-	EPEC	-	127	26	E2348/69
Escherichia	*coli*	-	EHEC	-	157	7	Sakai
Citrobacter	*rodentium*	-	-	-	152	-	ICC168

Table S2: List of Salmonella strains analyzed in the espJ homologue screen.

Species	Subspecies	Serovar	Serotype	Reference number	Origin	PCR *esp*J
S. enterica	*enterica*	Enteritidis	9,12:g,m:-	13015	Human faeces	-
				14142	Egg	-
		Typhimurium	4,5,12:i:1,2	13869	Human faeces	-
				12345	Veal	-
		Infantis	6,7:r:1,5	13243	Human faeces	-
				10098	Hen faeces	-
		Rissen	6,7:f,g:-	11921	Human faeces	-
				12247	Lamb	-
		Hadar	6,8:z10:e,n,x	10324	Human blood	-
				11801	Poultry	-
		Newport	6,8:e,h:1,2	11649	Human faeces	-
				12394	Water bath	-
		Derby	4,12:f,g:-	14241	Human faeces	-
				10675	Pork	-
		Montevideo	6,7:g,m,s:-	13593	Human faeces	-
				10535	Feed	-
		Virchow	6,7:r:1,2	13298	Human faeces	-
				13133	Poultry	-
		Ohio	6,7:b:l,w	12475	Human blood	-
				10595	Egg	-
		Altona	8,20:r:z6	13246	Human blood	-
				11850	Sewage water	-
		Anatum	3,10:l,v:1,5	14270	Human faeces	-
				14556	Sausage	-
		Pomona	28:y:1,7	13089	Human faeces	-
				12127	Turtle faeces	-
		Mikawasima	6,7:y:e,n,z15	13835	Human faeces	-
				12552	Feed	-
		Thompson	6,7:k:1,5	13024	Human faeces	-
				11612	Pork	-
S. enterica	*salamae*		4,12:b:-	13285	Human faeces	+
				12402	Water bath	+
			**13,22:z10:z6**	**13588**	**Water bath**	**+**
			28:z29:1,5	13042	Feed	-
			42:z:1,5	11465	Seafood	+
			53:d:z42	14256	Water bath	-
			6,8:m,t:1,6	12405	Water bath	-

S. enterica	*arizonae*	48:z4,z23:-	14016	Human faeces	+
			11134	Spring	+
			11144	Tap water	+
			12043	Human haeces	+
			12517	Water bath	+
			15264	River	+
			16166	Human urine	+
		42:r:-	13861	Human faeces	+
		35:z4,z23:-	14104	Feed	-
S. enterica	*diarizonae*	38:l,v:z35	10241	Human faeces	-
			11784	Seafood	-
		47:i:z53	15379	Human faeces	-
			10935	Pig	-
		47:c:1,5,7	14698	Seafood	-
		47:i:z	15355	Human faeces	-
			12377	Water bath	-
		35:z52:z35	13322	Human faeces	-
		47:k:z	12388	Water Bath	-
		47:k:z53	10934	Pig	-
		47:l,v:1,5,7	11375	Human faeces	-
		50:i:z	10592	Sewage water	-
		60:r:e,n,x,z15	15447	Human faeces	-
		60:z52:z53	10761	Human faeces	-
		65:k:z	14901	Human faeces	-
S. enterica	*houtanae*	45:g,z51	11818	Human faeces	-
		6,7:z36:-	13357	Human faeces	-
		44:z24,z23:-	10708	Human faeces	-
			14976	Human faeces	-
			14261	Water bath	-
		44:g,z51:-	14477	Human blood	-
S. bongori		60:z41	256		+
		61:z35	258		+
		40:z35	274		+
		48:i:-	275		+
		44:z39:-	276		+
		48:z35:-	277		+

Table S3: List of primers

#	Plasmid/Use	Primer Sequence (5'-3') (Restriction Sites Underlined)	Restriction site
1	*espJ* PCR screen	F-acgtcatatgcggtttcagaagg<br>R-ccggaagagttatgataaacggc	-<br>-
2	pCX340_SeoC (pICC2390)	F-ggaataacatatgaatgttataaagaactgtttttc<br>R-tctccgcggattttttacttacaggataaatatc	NdeI<br>SacII
3	pMXs-IP_GFP_FcγRIIa (pICC2437)	F-ggcctcgagatgtctcagaatgtatgtccc<br>R-caagcggccgctttacttgtacagctcgtccatg	XhoI<br>NotI
4	pMALXE_EHEC_EspJ (pICC2447)	F-cacggatccatgtcaattataaaaaactgcttatc<br>R-cacaagcttttatttttgagaggatatatgtcaac	BamHI<br>HindIII
5	pMALXE_*C. rodentium*_EspJ (pICC2448)	F-cacggatccccaattataaggtcctgtttatcatc<br>R-cacaagcttttattttttaaatgggtatatgtcaac	BamHI<br>HindIII
6	pMALXE_*S. salamae*_SeoC (pICC2449)	F-cacggatccaatgttataaagaactgtttttcatc<br>R-gataagcttctattttttacttacaggataaatatc	BamHI<br>HindIII
7	pMALXE_*S. arizonae*_SeoC (pICC2450)	F-cacggatccaatattatcaagaactgtttttcattcctcaac<br>R-gccaagcttctatttttgttcactggataaatatc	BamHI<br>HindIII
8	pMALXE_*S. bongori*_SboC (pICC2451)	F-cacggatccaatgttataaaaaaactgtctttcatc<br>R-cacaagcttctatttttgttcactggatagatatc	BamHI<br>HindIII
9	pRK5_*S. salamae*_SeoC (pICC2427)	F-ctgggatccatgaatgttataaagaactgtttttc<br>R-tggcggccaagcttctgcagctattttttacttacaggataaatatc	BamHI<br>PstI
10	pRK5_*S. arizonae*_SeoC (pICC2428)	F-cacggatccaatattatcaagaactgtttttcattcctcaac<br>R-gccaagcttctatttttgttcactggataaatatc	BamHI<br>HindIII
11	pRK5_S. *bongori*_SboC (pICC2429)	F-ctgggatccatgaatgttataaaaaaactgtctttc<br>R-gcttctgcagctatttttgttcactggatagatatc	BamHI<br>PstI
12	pRK5_*C. rodentium*_EspJ (pICC2426)	F-ctgggatccccaattataaggtcctgtttatc<br>R-gcttctgcagttattttttaaatgggtatatgtcaac	BamHI<br>PstI
13	pWSK29_*S. salamae*_SeoC (pICC2438)	F-tagtggatccaagaaggagatatacctacgtaatgaatgttataaagaactgtttttc<br>R-gataagcttctattttttacttacaggataaatatc	BamHI<br>HindIII
14	pWSK29_*S. salamae*_SeoC_R79A (pICC2466)	F-gcaattcaggaagataaatttacagatttaaaaag<br>R-tacagcaacaaatttttcagtaatattaaag	-<br>-
15	PCR amplification of *S. salamae* SeoC ± 500bp	F-aactttttgtgtaaattcttataaaacag<br>R- cgctcaatgcgggtattattc	-<br>-
16	Inverse PCR removal of SeoC from pGEMT	F-atatctctcatctggatggggac<br>R-ttttaactaatcctgcttaaaataatg	-<br>-
17	PCR check Kanamycin insert for *S. salamae* Δ*seoC*	F-catagtctgatactcttagatcatc<br>R-gttatttttcatggcgggaag	-<br>-
18	PCR amplification of kanamycin resistance cassette	F-catatgaatatcctccttag<br>R-tgtgtaggctggagctgcttcg	-<br>-
19	pMX-IP_GFP-FcγRIIa	F- ggcctcgagatgtctcagaatgtatgtccc<br>R- caagcggccgctttacttgtacagctcgtccatg	XhoI<br>NotI

#	Plasmid/Use	Primer Sequence (5'-3') (Restriction Sites Underlined)	Restriction site
20	PCR amplification of *S. salamae* EscN ± 50bp	F- gtttgattcctatcatgatctcattttggcatttttttctttatggccgtgtgtaggctggagctgcttcg<br>R- gcattaaagtcattaactgtccccaacacttttaattctatattattacccatatgaatatcctccttag	-<br>-
21	PCR amplification of *S. salamae* InvA ± 50bp	F- acttaacagtgctcgtttacgacctgaattactgattctggtactaatgggtgtaggctggagctgcttc<br>R- gctatctgctatctcaccgaaagataaaacctccagatccggaaaacgacccatatgaatatcctccttag	-<br>-
22	PCR amplification of *S. salamae* SsaV ± 50bp	F- gttcacgtttaggtgagagaatcagagcgcaacagtggctcaatgtatgctgtgtaggctggagctgcttcg<br>R- cctgaattcggccccatcgccgatagccatcgggggggaggatatttcagccatatgaatatcctccttag	-<br>-
23	PCR check Kanamycin insert for *S. salamae* Δ*escN*	F- caattaaccaagtctcaaaggc<br>R- gtacttttccccactccaga	-<br>-
24	PCR check Kanamycin insert for *S. salamae* Δ*invA*	F- gacgccagctgttcgc<br>R- caattccgcctcaataatgg	-<br>-
25	PCR check Kanamycin insert for *S. salamae* ΔssaV	F- ccagataactgttttaacgatgaa<br>R- gaactcgcggacttctcg	-<br>-
26	Src Y416F mutation by inverse PCR	F-ttcacagcacggcaaggtgc<br>R-ctcgttgtcctcgatgaggc	-<br>-
27	Src E310A mutation by inverse PCR	F-ggaggccctcctgcaggcacgccaagtg<br>R-cctcatcacttgggctgcctgcaggaag	-<br>-
28	$EspJ_{EPEC}$ R79A mutantion	F-tataaaacgggatttcgttgctgtagcaatccaaagtaatcagtttactgat<br>R-atcagtaaactgattactttggattgctacagcaacgaaatcccgttttata	-<br>-

Table S4: List of plasmids

Plasmid	Description	Source / Reference
pCX340	Expression vector for TEM-1 fusion protein,	(1)
pICC526	pCX340- expression of *S. bongori* SboI-TEM-1 fusion protein	(2)
pICC522	pCX340- expression of *S. bongori* FabI-TEM-1 fusion protein	(2)
pICC524	pCX340- expression of *S. bongori* SboC-TEM-1 fusion protein	(2)
pICC2390	pCX340- expression of *S. salamae* SeoC-TEM-1 fusion protein	This study
pRK5-myc	Eukaryotic expression vector of Myc tagged protein.	BD Pharmingen
pICC2271	pRK5- expression of myc tagged EPEC EspJ	(3)
pICC2304	pRK5- expression of myc tagged EHEC EspJ	This study
pICC2426	pRK5- expression of myc tagged *C.rodentium* EspJ	This study
pICC2427	pRK5- expression of myc tagged *S.salamae* SeoC	This study
pICC2428	pRK5- expression of myc tagged *S.arizonae* SeoC	This study
pICC2429	pRK5- expression of myc tagged *S.bongori* SboC	This study
pICC2275	pRK5- expression of myc tagged EPEC $EspJ_{R79A/D187A}$	(3)
pMALXE	Expression MBP tagged fusion proteins in *E. coli* expression host	(4)
pICC2446	pMALXE- expression of MBP tagged EPEC EspJ	This study
pICC2447	pMALXE—expression of MBP tagged EHEC EspJ	This study
pICC2448	pMALXE- expression of MBP tagged *C.rodentium* EspJ	This study
pICC2449	pMALXE - expression of MBP tagged *S.salamae* SeoC	This study
pICC2450	pMALXE - expression of MBP tagged *S.arizonae* SeoC	This study
pICC2451	pMALXE - expression of MBP tagged *S.bongori* SboC	This study
pICC2453	MALXE- expression of MBP tagged EPEC $EspJ_{R79A}$	This study
pGEX-KG	Expression GST tagged fusion proteins in *E. coli* expression host	GE Healthcare
pICC2458	pGEX-KG– expression of GST tagged $Src_{250\text{-}533\text{-}K295M/Y416A}$	This study
pICC2486	pGEX-KG– expression of GST tagged $Src_{250\text{-}533\text{-}K295M/Y416F/E310A}$	This study
pEGFP-FcγRIIa	Expression of EGFP tagged FcγRIIa in mammalian host	(5)
pMXs-IP	Retroviral plasmid for viral package signal, transcription and processing, puromycin selectable	Invitrogen
pICC2437	pMXs-IP-GFP-FcγRIIa	This study
pCMV-VSV-G env	Envelope glycoprotein of Vesicular stomatis virus	Walther Mothes, Yale University
pCMV-MMLV-gag-pol	Capsid, reverse transcriptase and insertase	Walther Mothes, Yale University

pWSK29	Expression vector for bacteria	(6)
pICC2438	pWSK29 - expression of *S. salamae seoC*	This study
pICC2466	pWSK29 - expression of *S. salamae seoC*$_{R79A}$	This study
pKD46	Arabinose inducible expression of λ-red recombinase	(7)
pKD4	Kanamycin resistance cassette	(7)
pGEMT	High copy cloning plasmid	Promega
pICC2439	pGEMT-Kn resistance cassette flanked by 500 bp up/downstream of *S. salamae* SeoC coding sequence—for knocking out SeoC on chromosome	This study
pFPV25.1	Constitutive GFP expression used for visualisation of *Salmonella* during infection	(8)

Table S5: Primary and secondary antibodies, and reagents

Antibody/Reagent	Primary/ Secondary	Fluorophore	Species	Source	Use (Dilution)
α-myc	Primary	-	Chicken	Bethyl-laboratories Inc.	IF (1:100)
α-CSA-1	Primary	-	Goat	Insight biotechnology	IF (1:200)
α-BSA	Primary	-	Mouse	Sigma-Aldrich	IF/Opsonisation (1:100)
α-GST	Primary	-	Mouse	Abcam	WB (1:2000)
α-MBP-HRP	Conjugate	-	Mouse	Abcam	WB (1:2000)
α-Mouse	Secondary	RRX	Donkey	Jackson-Immunoresearch	IF (1:500)
α-Mouse	Secondary	AMCA	Donkey	Jackson-Immunoresearch	IF (1:50)
α-Chicken	Secondary	Alexa 647	Goat	Jackson-Immunoresearch	IF (1:100)
α-Mouse	Secondary	HRP	Goat	Jackson-Immunoresearch	WB (1:10000)
α-Mouse	Secondary	Alexa 488	Donkey	Jackson-Immunoresearch	IF (1:200)
α-Goat	Secondary	RRX	Donkey	Jackson-Immunoresearch	IF (1:200)
Phalloidin iFluor-647	Conjugate	Alexa 647	-	AAT Bioquest	IF (1:10000)
Streptavidin-HRP	Conjugate	-	n/a	Dako	WB (1:5000)

Table S6: Percentage sequence identity between SeoC/SboC/EspJ

**Full 217 residues**	**EPEC**	**EHEC**	***C. rodentium***	***S. salamae***	***S. arizonae***
**EHEC**	83				
C. rodentium	83	92			
S. salamae	58	57	56		
S. arizonae	57	57	56	83	
S. bongori	57	57	56	78	77
**N-terminal 50 residues**	**EPEC**	**EHEC**	***C. rodentium***	***S. salamae***	***S. arizonae***
**EHEC**	69				
C. rodentium	65	80			
S. salamae	35	37	35		
S. arizonae	31	35	31	71	
S. bongori	35	41	35	80	63

References

1. **Charpentier X**, **Oswald E**. 2004. Identification of the secretion and translocation domain of the enteropathogenic and enterohemorrhagic Escherichia coli effector Cif, using TEM-1 beta-lactamase as a new fluorescence-based reporter. J Bacteriol **186**:5486–95.
2. **Fookes M**, **Schroeder GN**, **Langridge GC**, **Blondel CJ**, **Mammina C**, **Connor TR**, **Seth-Smith H**, **Vernikos GS**, **Robinson KS**, **Sanders M**, **Petty NK**, **Kingsley RA**, **Bäumler AJ**, **Nuccio S-P**, **Contreras I**, **Santiviago CA**, **Maskell D**, **Barrow P**, **Humphrey T**, **Nastasi A**, **Roberts M**, **Frankel G**, **Parkhill J**, **Dougan G**, **Thomson NR**. 2011. Salmonella bongori provides insights into the evolution of the Salmonellae. PLoS Pathog **7**:e1002191.
3. **Young JC**, **Clements A**, **Lang AE**, **Garnett JA**, **Munera D**, **Arbeloa A**, **Pearson J**, **Hartland EL**, **Matthews SJ**, **Mousnier A**, **Barry DJ**, **Way M**, **Schlosser A**, **Aktories K**, **Frankel G**. 2014. The Escherichia coli effector EspJ blocks Src kinase activity via amidation and ADP ribosylation. Nat Commun **5**:5887.
4. **Moon AF**, **Mueller GA**, **Zhong X**, **Pedersen LC**. 2010. A synergistic approach to protein crystallization: combination of a fixed-arm carrier with surface entropy reduction. Protein Sci **19**:901–13.
5. **van Zon JS**, **Tzircotis G**, **Caron E**, **Howard M**. 2009. A mechanical bottleneck explains the variation in cup growth during FcgammaR phagocytosis. Mol Syst Biol **5**:298.
6. **Wang RF**, **Kushner SR**. 1991. Construction of versatile low-copy-number vectors for cloning, sequencing and gene expression in Escherichia coli. Gene **100**:195–9.
7. **Datsenko KA**, **Wanner BL**. 2000. One-step inactivation of chromosomal genes in Escherichia coli K-12 using PCR products. Proc Natl Acad Sci U S A **97**:6640–5.

8. **Valdivia RH**. 1997. Fluorescence-Based Isolation of Bacterial Genes Expressed Within Host Cells. Science **277**:2007–2011.
